# Recommendations for the diagnosis and treatment of alpha-1 antitrypsin deficiency

**DOI:** 10.36416/1806-3756/e20240235

**Published:** 2024-11-16

**Authors:** Paulo Henrique Ramos Feitosa, Maria Vera Cruz de Oliveira Castellano, Claudia Henrique da Costa, Amanda da Rocha Oliveira Cardoso, Luiz Fernando Ferreira Pereira, Frederico Leon Arrabal Fernandes, Fábio Marcelo Costa, Manuela Brisot Felisbino, Alina Faria França de Oliveira, Jose R Jardim, Marc Miravitlles

**Affiliations:** 1. Hospital Regional da Asa Norte, Brasília (DF) Brasil.; 2. Hospital do Servidor Público Estadual de São Paulo - IAMSPE - São Paulo (SP) Brasil.; 3. Universidade do Estado do Rio de Janeiro - UERJ - Rio de Janeiro (RJ) Brasil.; 4. Hospital das Clínicas, Universidade Federal de Goiás - HU-UFG - Goiás (GO) Brasil.; 5. Hospital das Clínicas - Universidade Federal de Minas Gerais - UFMG - Belo Horizonte ( MG) Brasil.; 6. Divisão de Pneumologia, Instituto do Coração, Hospital das Clínicas, Faculdade de Medicina, Universidade de São Paulo, São Paulo ( SP) Brasil.; 7. Complexo Hospital das Clínicas, Universidade Federal do Paraná - CHC-UFPR - Curitiba (PR) Brasil.; 8. Hospital Universitário, Universidade Federal de Santa Catarina - HU-UFSC - Florianópolis (SC) Brasil.; 9. Hospital Otávio de Freitas, Secretaria de Saúde do Estado de Pernambuco, Recife (PE) Brasil.; 10. Universidade Federal de São Paulo, São Paulo (SP) Brasil.; 11. Vall d’Hebron Institut de Recerca - VHIR - Hospital Universitário Valld’Hebron, Barcelona, España.

**Keywords:** alpha 1-antitrypsin, Emphysema, Pulmonary disease, chronic obstructive, alfa 1-antitripsina, Enfisema, Doença pulmonar obstrutiva crônica

## Abstract

Alpha-1 antitrypsin deficiency (AATD) is a relatively rare genetic disorder, inherited in an autosomal codominant manner, that results in reduced serum AAT concentrations, with a consequent reduction in antielastase activity in the lungs, as well as an increased risk of diseases such as pulmonary emphysema, liver cirrhosis, and necrotizing panniculitis. It results from different mutations in the SERPINA1 gene, leading to changes in the AAT glycoprotein, which can alter its concentration, conformation, and function. Unfortunately, underdiagnosis is quite common; it is possible that only 10% of cases are diagnosed. The most common deficiency is in the Z variant, and it is estimated that more than 3 million people worldwide have combinations of alleles associated with severe AATD. Serum AAT concentrations should be determined, and allelic variants should be identified by phenotyping or genotyping. Monitoring lung function, especially through spirometry, is essential, because it provides information on the progression of the disease. Although pulmonary densitometry appears to be the most sensitive measure of emphysema progression, it should not be used in routine clinical practice to monitor patients. In general, the treatment is similar to that indicated for patients with COPD not caused by AATD. Exogenous administration of purified human serum-derived AAT is the only specific treatment approved for AATD in nonsmoking patients with severe deficiency (serum AAT concentration of < 57 mg/dL or < 11 µM), with evidence of functional loss above the physiological level.

## RESUMO

A deficiência de alfa-1 antitripsina (DAAT) é uma herança genética autossômica codominante, relativamente rara, que resulta em concentração reduzida de alfa-1 antitripsina (AAT) no soro e, portanto, redução na atividade antielastase nos pulmões e aumento do risco de enfisema pulmonar, cirrose hepática e paniculite necrotizante. Resulta de diferentes mutações no gene SERPINA1 levando a mudanças na glicoproteína AAT, que podem alterar a sua concentração, conformação e/ou função. Infelizmente, o subdiagnóstico é muito comum, e é possível que apenas 10% dos casos estejam diagnosticados. A variante Z é a deficiência mais comum, e estima-se que mais de 3 milhões de pessoas em todo o mundo tenham combinações de alelos associadas à deficiência grave de AAT. É necessária a determinação da concentração sérica de AAT e a identificação de variantes alélicas por fenotipagem ou genotipagem. É fundamental o acompanhamento da função pulmonar, principalmente por espirometria, pois essa informa sobre a progressão da doença. A densitometria pulmonar parece ser a medida mais sensível da progressão do enfisema, mas não deve ser usada no acompanhamento de pacientes na prática clínica de rotina. O tratamento geral é semelhante ao indicado para pacientes com DPOC não causada por DAAT. A administração exógena de AAT derivada de soro humano purificado é o único tratamento específico aprovado para DAAT em pacientes não fumantes e com deficiência grave (nível sérico < 57 mg/dL ou AAT sérica < 11 μM), com comprovação de perda funcional acima da fisiológica.

## INTRODUCTION

Alpha-1 antitrypsin deficiency (AATD) is a genetic disorder that was first recognized in 1963. Underdiagnosis of AATD has been widely reported in the literature.^1^ It is a relatively rare genetic disorder that is inherited in an autosomal codominant manner and results in reduced serum AAT concentrations, consequently reducing antielastase activity in the lungs, as well as increasing the risk of diseases such as pulmonary emphysema, liver cirrhosis, and necrotizing panniculitis.^2^ ^,^ ^3^

The serine protease inhibitor gene (*SERPINA1*), located on chromosome 14q32.1, encodes AAT. This gene is highly pleomorphic and its variants can be related to normal or below-normal concentrations of AAT in mild, moderate, or severe forms, or even to a total lack of AAT secretion (undetectable serum concentrations), designated the null variant.^4^ In most countries, the prevalence of AATD is not known, being better known in Europe and the United States.^5^ There is now more information on the disorder, and tests made available by the pharmaceutical industry have facilitated the genetic diagnosis.^6^ ^,^ ^7^

Although AAT production occurs mainly in hepatocytes, it also occurs in intestinal cells, alveoli, macrophages, neutrophils, and the cornea.^3^ The protein is released into the bloodstream and acts as a protector against neutrophil elastase, mainly in the lungs.^3^ Its half-life is 3-5 days, and serum concentrations vary little in normal individuals, although they can be elevated during inflammatory processes.^3^

Unfortunately, it is possible that only 10% of cases of AATD are diagnosed, a high rate of underdiagnosis that precludes genetic counseling and makes appropriate treatment difficult.^8^ The determination of serum concentrations of AAT is recommended in all patients with COPD, liver disease, necrotizing panniculitis, granulomatosis with polyangiitis, or bronchiectasis without a defined cause.^2^ ^,^ ^9^ The World Health Organization (WHO) suggests that patients diagnosed with late-onset asthma be evaluated for AATD.^10^

Patients with pulmonary manifestations should be monitored according to the recommendations for COPD.^11^ In some cases, such patients should be referred for specific treatment with AAT replacement.^12^

The aims of this article are to improve care for individuals with AATD; to accurately inform fellow pulmonologists about AAT and AATD; to promote active AATD case finding; to encourage the creation of treatment guidelines; to stimulate the evaluation of AAT in all diseases potentially associated with AATD; and to investigate the family members of patients with AATD, as well as individuals with suspicious findings on imaging examinations and those with clinical symptoms or functional alterations. Thus, the rates of underdiagnosis could be reduced and the possibility of appropriate care could be increased.

## GENETICS

Mutations in the *SERPINA1* gene lead to different changes in the AAT glycoprotein, which can alter its concentration, conformation, and function.^5^ ^,^ ^13^ More than 200 variants of the *SERPINA1* gene have been described.^14^ The AAT variants initially found were classified with letters from A to Z, depending on the speed of migration of the molecule in a pH gradient after isoelectric focusing. The normal protease inhibitor (PI) of AAT is designated with the letter M, also called the M allele, and is present in 85-90% of the population.^13^ ^,^ ^15^ Individuals homozygous for the M allele are designated Pi*MM homozygotes and have normal serum AAT concentrations (100-220 mg/dL). The M allele has some benign subvariants, including two M1 variants (Ala213 and Val213), as well as variants M2, M3, and M4, and there are tests that can be employed to differentiate among those.^16^ Other benign alleles are E, G, and Zpratt, which encode AAT variants that do not decrease the serum concentration or function of the protein.^17^ ^,^ ^18^

The allele most commonly associated with severe AATD is Z, mainly in homozygosity (Pi*ZZ), presenting serum AAT concentrations of 10-20% of normal (20-45 mg/dL). The Z allele is found most commonly in northern and western Europe.^5^ ^,^ ^16^ ^,^ ^19^ In the Z allele, glutamic acid is replaced by lysine at position 342 of the *SERPINA1* gene, and this mutation leads to the synthesis of a malformed protein, which can polymerize and accumulate in hepatocytes, potentially causing liver disease.^5^ ^,^ ^15^ Subsequently, the secretory defect leads to a reduction in serum concentrations and activity, predisposing to the onset of emphysema.^5^ ^,^ ^20^ ^,^ ^21^ Among the known mutations, Pi*ZZ is present in 95% of cases of severe deficiency and the Z allele can be found in one in 25 people of European ancestry. Heterozygous mutations, such as Pi*MZ, generally cause a mild to moderate reduction in AAT. Combinations of the Z allele and other pathogenic variants (e.g., Pi*SZ and M_Procida_) can result in below-normal serum AAT concentrations and lung disease.^5^ ^,^ ^15^ However, there are variations in the clinical expression of the disease, in which the presence of AATD alone is not sufficient to induce pulmonary emphysema. Factors such as smoking, environmental exposure, and genetic factors predispose to the onset of emphysema in these cases.^9^ ^,^ ^22^ Many patients with the Pi*ZZ genotype will never develop pulmonary emphysema over the course of their lives.

Other alleles associated with severe AATD are M_Procida_, M_Heerlen_, M_Malton_, S_Iiyama_, P_Lowell_, as well as some rarer variants and the family of null (Q0) alleles, which do not produce AAT.^5^ ^,^ ^15^ Alleles such as the variants I and S, the latter being the variant most commonly found in Mediterranean Europe (mainly in the Iberian Peninsula), lead to lower serum concentrations than in the Pi*MM genotype, although still with protective value, resulting in a clinical form of AATD that is milder.^5^ However, when these variants are associated with risk factors, such as smoking, they can lead to pulmonary involvement.^2^ ^,^ ^23^ The so-called dysfunctional variants, including the F and Pittsburgh variants, lead to the production of AAT with abnormal function, with low binding to neutrophil elastase or altered inhibitory activity.^23^ ^,^ ^24^

The Z, S_Iiyama_, M_Malton_, and King alleles do not affect synthesis, although 70% of the mutant AAT is retained within the hepatocyte and 15% forms polymers that are not fully degraded and accumulate in the liver, causing chronic disease. The formation of hepatic polymers is directly related to structural alterations in the mutant protein.^25^ Currently, it is known that several other alleles, although considered rare, can cause serious liver problems^12^: Pi*M_Palermo_, Pi*M_Nichinan_, Pi*P_Lowell_, Pi*P_Duarte_, Pi*Q0_Cardiff_, Pi*Y_Barcelona_, and Pi*Z_Augsburg_.

The alleles S, I, and Queen can also form polymers, although at a slower rate, facilitating their removal and rarely causing liver injury (Chart 1). In addition, higher serum concentrations of AAT are released, generally conferring protection of the lungs in nonsmokers.^3^

**Chart 1:** Major alleles associated with alpha-1 antitrypsin deficiency.^3^ ^,^ ^15^

Allele	Clinical significance
F	Uncertain; slight reduction in activity
I	Uncertain; slight reduction in activity
S	Uncertain; slight reduction in activity
Z	Marked reduction in activity; risk of emphysema and liver disease
M_Procida_	Marked reduction in activity; risk of emphysema
M_Malton_	Marked reduction in activity; risk of emphysema and liver disease
S_Iiyama_	Marked reduction in activity; risk of emphysema and liver disease
P_Lowell_	Marked reduction in activity; risk of emphysema and liver disease
M_Heerlen_	Marked reduction in activity; risk of emphysema
Q0_Granite Falls_	No protein expression; risk of emphysema
Q0_West_	No protein expression; risk of emphysema
Q0_Bellingham_	No protein expression; risk of emphysema
Q0_Mattawa_	No protein expression; risk of emphysema

## EPIDEMIOLOGY

The prevalence of AATD has not yet been defined in most countries, although it is known to vary across geographic regions and racial groups and to primarily affect White individuals of European descent.^19^ It is estimated that more than 3 million people worldwide have allele combinations associated with severe AATD^26^ and that the Pi*ZZ genotype occurs in 1:2,000-5,000 individuals in Europe and in 1:5,000-7,000 individuals living in countries with European immigration, such as the United States and Australia.^19^ Although little is known about the prevalence of the most common rare alleles, even less is known about the rare non-S and non-Z alleles. A review of the Spanish Registry of AATD patients from 1998 to 2010 revealed that 56 (1.6%) of 3,511 patients with AATD had rare alleles.^27^

Registries such as that of the Alpha-1 Foundation in the United States and, more recently, that of the European Alpha-1 Research Collaboration (EARCO) are very important. Such registries allow us to understand AATD in a given location, to characterize patients, and to establish programs aimed at early diagnosis, as well as enabling the expansion of knowledge about the natural history of the disease. Between 2020 and 2022, the EARCO evaluated 1,044 individuals with AATD (defined as a serum AAT concentration < 11 µM, or 50 mg/dL) in 15 countries.^28^ Among the 629 individuals (60.2%) who had the Pi*ZZ genotype, most (51.5%) were male; the mean age was 55.6 ± 13.2 years; the mean age at diagnosis was 44.7 ± 16.7 years; the mean FEV_1_ was 66.9 ± 30.7%; the mean DL_CO_ was 68.0 ± 23.2%; the mean COPD Assessment Test score was 13.2 ± 9.3 points; and 190 (30.2%) had received AAT replacement therapy.

There are several ways to assess the prevalence of a genetic disease: screening newborn infants; measuring the concentration in blood stored at a blood bank; and evaluation in genetic databases or in the general population. In Sweden, 200,000 infants born between 1972 and 1974 were evaluated and the Pi*ZZ genotype was found in 127 (0.06%), or one in every 1,600.^5^ Although screening neonates allows guidance to be given to avoid exposure to risk factors such as smoking and environmental or occupational pollution, ethical aspects have made such screening unfeasible. Of 20,000 blood donors in the United States in 1988, 2,850 (0.03%) were found to have the Pi*ZZ genotype.^5^

It is also possible to track AATD cases by evaluating patients with diseases caused by AATD. Among patients with COPD, it is expected that 1-4% will be found to have the Pi*ZZ genotype, which is why the WHO recommends AAT testing for all patients diagnosed with COPD.^10^ Recently, the Pi*ZZ/COPD prevalence ratio in Europe was reported to be 0.12% (0.08-0.24%), with considerable differences among countries.^19^ In Argentina, the prevalence of AATD among patients with COPD was found to be 0.83%.^29^ In Brazil, a cross-sectional study evaluated 926 patients with COPD in five different states; the authors found that 0.8% of the patients had the Pi*ZZ genotype and that 2.80% had some variant allele, although only the S and Z variants were evaluated.^30^

In a recent study, the frequency of variant alleles was described in 30,827 individuals who might have some deficiency were evaluated-by buccal swab or by dried blood spot (DBS) sampling (collection of a blood drop on filter paper)-between 2018 and 2022 in six countries (Argentina, Brazil, Chile, Colombia, Spain, and Turkey). The prevalence was found to be 12.7% for the MS genotype, whereas it was 7.4%, 3.0%, 1.6%, and 0.8% for the MZ, ZZ, SZ, and SS genotypes, respectively.^7^ In another analysis of that sample of individuals,^6^ all variant alleles were evaluated and mutations were found in 9,528 (30.9%), of whom 818 (2.7%) had rare alleles (excluding all S and Z alleles). The authors stated that the identification of the range of alleles can establish a new distribution of alleles in various countries and that their findings can facilitate the selection of new alleles to include in the diagnostic panel.

## RISK FACTORS FOR LUNG DISEASE

As demonstrated in previous studies,^3^ ^,^ ^31^ ^,^ ^32^ AATD is a heterogeneous condition in which the combination of the genotype with low serum AAT concentration and risk factors are fundamental for the emergence and progression of its clinical and functional manifestations. Although smoking continues to be the main risk factor for lung disease, other factors, including predisposing familial conditions, respiratory infections, and exposure to environmental or occupational pollutants, which also increase inflammation and elastase in the airways and alveoli, have increasingly been studied and valued.^31^ ^-^ ^33^

Individuals with undetectable AAT associated with null alleles can develop emphysema, even without a history of smoking, as can those with marked deficiency (AAT concentrations < 11 µM) associated with Z alleles, homozygous Pi*ZZ genotypes, or rare variants.^3^ ^,^ ^9^ However, exposure to tobacco smoke increases the number of neutrophils and macrophages in the lungs and increases the individual production of elastase per cell, accelerating the onset, severity, and progression of emphysema in these individuals. The progression of emphysema is related to the age at which smoking began, as well as to the lifetime smoking history.^9^ ^,^ ^34^ ^,^ ^35^

In a registry of patients with AATD in follow-up for 11 years in Germany, there was an association between a greater decline in FEV_1_ and a shorter time since smoking cessation, occupational exposure to inhalants, and the frequency of COPD exacerbations.^36^ In a study of smokers between 37 and 39 years of age with the Pi*ZZ genotype and normal spirometry results, it was observed that there could be changes in DL_CO_ and small airway disease, consistent with emphysema.^37^ In a study of patients with the Pi*ZZ genotype, a high level of exposure to environmental pollution was associated with worsening gas exchange and respiratory status.^38^

Heterozygous individuals, especially those with the Pi*MZ and Pi*SZ alleles, can also develop emphysema depending on the degree of exposure to smoking; although that risk is low in nonsmokers, it is still a matter of controversy.^31^ ^,^ ^39^ In a study of smokers with AATD,^31^ the risk of COPD was found to be 5.2 times higher among those with the Pi*MZ genotype than among those with the Pi*MM genotype.^31^ However, many studies that have demonstrated greater loss of lung function in individuals with the Pi*MZ genotype are biased by not having adjusted that loss for smoking history. When thus adjusted, the decline in FEV_1_ is generally small.

One study demonstrated that the risk of developing emphysema among smokers with the Pi*SZ genotype was similar to that among smokers with the Pi*MM genotype, although those in the former group had sought medical evaluation earlier.^39^ In another study*,* never-smokers with the Pi*SZ genotype did not present an increased risk of developing COPD, whereas smokers with the same genotype had greater airflow obstruction and former smokers with the genotype did not have a greater decline in FEV_1_.^40^ Currently, there is concern about individuals carrying the Pi*SZ alleles, who should be advised not to expose themselves to risk factors and to have periodic follow-up examinations.

In the last decade, with the new taxonomy and etiopathogenesis of COPD, early-life risk factors, such as prematurity, low birth weight, and exposure to smoking (in intrauterine life and infancy), as well as asthma, infections, and environmental/occupational exposures throughout life, have been given ever greater weight.^11^ ^,^ ^41^ The influence of many of these risk factors for bronchopulmonary disease needs to be better studied in AATD, not only in relation to its early onset but also in relation to its progression. In COPD, especially in smokers with COPD, airflow obstruction is caused by inflammation in the small airways and loss of elastic recoil due to emphysema, changes that cannot be identified by spirometry.^11^ ^,^ ^41^

Although the role of small airway disease as a risk factor, early marker of lung involvement, prognostic factor, and treatable feature in patients with AATD was well discussed in a recent review,^42^ further studies are needed. A study of 193 patients with AATD evaluated nitrogen washout, a sensitive method for identifying small airway disease, and showed that the lung clearance index *was* abnormal in 83% of the patients with the Pi*ZZ genotype, in 47% of those with other genotypes, and in 43% of the 117 patients with normal FEV_1_.^43^

The Subpopulations and Intermediate Outcome Measures in COPD Study showed that smokers with the Pi*ZZ or Pi*MZ genotype had more bronchiectasis than did those with other genotypes, and that those changes were associated with a greater degree of emphysema, more small airway disease, and more severe clinical repercussions than what was seen in smokers without bronchiectasis.^44^

The relationship between asthma and AATD remains controversial, and although some studies have shown the prevalence of asthma to be higher among patients with AATD, that finding is usually concomitant with a diagnosis of COPD.^33^ In principle, asthma is not a risk factor for an accelerated decline in lung function, and AAT replacement does not prevent a loss of function due to asthma.^33^

## PULMONARY CLINICAL MANIFESTATIONS

The classic clinical presentation of AATD is the early onset of COPD. Considerable variability in the time to symptom onset has been described, although it rarely appears before 25 years of age. Symptoms that are more severe are more commonly seen in smokers and former smokers, which also influences the mean age at symptom onset, which is typically 32-40 years in smokers and 48-54 years in nonsmokers.^45^ ^-^ ^47^

The characteristics that distinguish AATD-related COPD in nonsmokers or smokers without occupational risk factors from COPD caused by smoking (unrelated to AATD) are the early onset of symptoms, panacinar emphysema, and a predominance of radiological changes at baseline.^5^ However, the “classic” presentation of smoking-related COPD also occurs in patients with AATD and should not rule out the possibility in such patients.^47^ ^,^ ^48^ It is important to emphasize that up to 37% of individuals with severe AATD have emphysema predominantly in the upper lobes.^19^ ^,^ ^49^

Among the most common symptoms of AATD,^50^ dyspnea on exertion is the most prevalent (in 84% of cases), followed by wheezing associated with respiratory infections (in 76%); wheezing without respiratory infections (in 65%); cough and phlegm (in 50%); and chronic cough (in 42%). On spirometry, obstructive ventilatory defect is a classical finding, with normal or reduced FVC. In the analysis of lung volumes, RV and TLC can be increased, with reduced DL_CO_. A response can be seen after bronchodilator use, and concomitance with asthma can be associated with faster progression of the disease.^51^ Factors associated with a rapid decline in lung function include symptom onset between 30 and 44 years of age, male gender, low BMI, low serum AAT concentrations, frequent exacerbations, reversibility by bronchodilators, and a severe reduction in functional capacity.^52^ ^,^ ^53^ Although the association between AATD and bronchiectasis is known, the pathophysiology of the onset of the symptoms has yet to be well defined. Its prevalence varies greatly among studies, ranging from 26% to 52% in the studies with the largest patient samples, although the presence of bronchiectasis without emphysema should not rule out a diagnosis of AATD.^54^

## EXTRAPULMONARY CLINICAL MANIFESTATIONS

### Liver disease

Liver disease is the second most common manifestation of AATD in adults and the second leading cause of death in patients with the disorder.^55^ Liver disease associated with AATD occurs primarily due to abnormal accumulation of mutated protein polymers within the rough surface of the endoplasmic reticulum of hepatocytes. These polymers can lead to inflammation, with consequent liver fibrosis, cirrhosis, and increased risk of hepatocellular carcinoma, especially in individuals with hepatitis B.^55^ Because liver disease is highly variable and not all patients with the ZZ genotype develop the disease despite the presence of polymers in the liver, there must be other causal factors that are not yet fully understood. It has been suggested that genetic determinants of intracellular protein processing have some influence on susceptibility to liver disease in AATD.^56^ Patients with genetic variants associated with the presence of hepatic polymers, such as S_Iiyama_, M_Duarte_, M_Malton_, and particularly the presence of the Z allele, can develop signs of liver disease.^56^

Liver disease has a bimodal age distribution, with the first peak in early childhood and the second in individuals over 50 years of age.^57^ In the neonatal period, prolonged cholestasis is the main clinical manifestation. A study that evaluated 200,000 newborns in Sweden identified the Pi*ZZ genotype in 127, of whom 73% had prolonged jaundice and 8% had severe liver disease.^58^ Alterations in liver enzymes were identified in 50% of those neonates, with spontaneous resolution within months. At 18 years of age, most were healthy. Only 3% had severe progressive disease.^59^ In the pediatric population, AATD accounts for 3.5% of the indications for liver transplantation.^60^ In adults, liver fibrosis occurs in 20-36% of patients with the Pi*ZZ genotype, and advanced fibrosis is 10-20 times more common in such patients than in those with other genotypes.^61^ Approximately 10% of patients with severe AATD develop cirrhosis, and 14.5% of such patients require liver transplantation.^60^ ^,^ ^62^ Only a small percentage of patients with AATD and advanced liver fibrosis present alterations in liver enzymes, with levels varying over the years.^63^

In patients with AATD, gamma-glutamyl transferase is the most sensitive marker for the diagnosis of liver disease, with a mean concentration significantly higher than that of glutamic pyruvic transaminase.^64^ Although patients with the Pi*SS genotype can have mild transaminase alterations, they do not develop hepatobiliary disease.^64^ Patients with the Pi*SZ genotype show elevated liver enzymes, with a clear predisposition to fibrosis, liver cirrhosis, and hepatocellular carcinoma, although that predisposition is much lower than that observed in patients with the Pi*ZZ genotype.^57^ ^,^ ^64^ Patients with the Pi*MS genotype do not show an increased risk of liver disease.^64^

Noninvasive liver evaluation through biochemical studies, transient hepatic elastography, and the determination of phenotypes/genotypes should be performed routinely in individuals with AATD.^5^ Given the nature and risk of complications, liver biopsy is reserved for selected cases only. Although transient hepatic elastography is useful to rule out advanced fibrosis (stages 3 and 4), it is less effective in the early stages.^62^ In patients with altered enzymes, cirrhosis, or portal hypertension, liver ultrasound every six months is indicated in order to screen for hepatocellular carcinoma.^65^

There are no currently approved therapies for liver disease other than liver transplantation in patients with advanced disease.^66^ Promising strategies are being investigated, such as small RNA molecules that block polymer formation or stimulate pathways that accelerate their elimination. Fazirsiran, an RNA interference therapeutic that targets AAT and Z-AAT messenger RNA for degradation, was evaluated in a small phase 2 study of 16 patients with the Pi*ZZ genotype and was found to provide an 83% decrease in total liver Z-AAT after 24-48 weeks and improvements in histological liver abnormalities, including a reduction in the portal inflammation score in two thirds of the patients.^67^

### Panniculitis

Panniculitis associated with AATD is an extremely rare disease, and its clinical manifestations include nodular, painful, red skin lesions, often with an oily discharge, affecting areas of previous trauma such as the thighs, buttocks, abdomen, and upper limbs.^68^ It affects men and women with equal frequency, and the frequency also does not vary among the various phenotypes. It is much more associated with the Pi*ZZ genotype, and its pathogenesis is related to the lack of any opposition to proteolytic activity in the subcutaneous fat tissue.^68^ A number of therapies, including antibiotics, anti-inflammatory agents, and chemotherapeutics, have been tested, with varying rates of success. Treatment with AAT replacement has shown excellent results, with rapid clinical responses, and should be used in cases refractory to other therapies.^69^

### Associations with other diseases

There is insufficient evidence to support a relationship between AATD and other diseases (vascular disease, inflammatory bowel disease, glomerulonephritis, and systemic vasculitis). It has been suggested that patients with the Pi*ZZ genotype are susceptible to several vascular abnormalities, including abdominal/intracranial aneurysms, arterial fibromuscular dysplasia, and venous thromboembolism, on the basis of the principle that unopposed proteolytic activity damages vessel walls in severely deficient individuals.^5^ ^,^ ^70^ ^,^ ^71^ One report described the case of an individual with the Pittsburgh AAT mutation who died of severe bleeding after a viral infection.^72^ There is a functional relationship between the plasma proteins AAT and antithrombin III. To elucidate this relationship, the plasma of a 14-year-old boy who died of a hemorrhagic disorder was studied, and a variant of AAT was found in which the methionine at position 358 was replaced by an arginine, converting the normal function of AAT as an elastase inhibitor to that of a thrombin inhibitor.^72^ The data regarding the association between AATD and inflammatory bowel disease are conflicting and cannot be legitimized. One study evaluated children with the Pi*ZZ genotype who developed significant liver disease and associated nephropathy.^73^ In patients with AATD, there appears to be an increased risk of cytoplasmic antineutrophil cytoplasmic antibody (C-ANCA)-associated vasculitis, which is supported by plausible pathogenetic mechanisms. In the extravascular fluid, AAT plays an important role as an inhibitor of proteinase 3, a serine protease similar to neutrophil elastase and located in the primary granules of neutrophils. If left unchecked, proteinase 3 has potent tissue-destructive capacity, and AATD could therefore trigger an autoimmune response, allowing increased extracellular exposure to proteinase 3.^74^

## DIAGNOSIS

### When to test

A diagnosis of AATD has several immediate impacts,^75^ such as the need to test family members; the opportunity to implement educational measures; early intervention regarding smoking habits; advising patients about environmental and occupational exposures; and the opportunity to consider specific AATD treatment. The WHO recommends AAT testing in all patients diagnosed with COPD or adult-onset asthma.^10^ The GOLD and international AATD guidelines recommend that AATD screening be performed at least once in all individuals with COPD, regardless of age, ethnicity, or disease severity,^11^ as well as in those with emphysema or asthma with fixed airway obstruction.^2^ ^,^ ^5^ ^,^ ^9^ ^,^ ^76^ ^-^ ^78^ Despite global efforts to recommend AATD screening, the condition is still highly underdiagnosed.^79^ In most individuals with AATD, there is a long interval between symptom onset and the diagnosis of the disorder.^80^ As detailed in Chart 2, all individuals with chronic liver disease of undetermined etiology should also be screened for AATD, as should individuals with necrotizing panniculitis, C-ANCA-positive vasculitis, bronchiectasis of undetermined etiology, or a family history of any of the above.^2^ ^,^ ^5^ ^,^ ^77^

**Chart 2:** Indications for evaluation of alpha-1 antitrypsin deficiency.

• COPD or emphysema (regardless of age or ethnicity) • Adult-onset asthma or asthma with fixed airway obstruction • Bronchiectasis of undetermined etiology • Chronic liver disease of undetermined etiology • Necrotizing panniculitis • C-ANCA positive vasculitis, including granulomatosis with polyangiitis • First-degree relatives and partners of individuals with AATD* • Family history of emphysema, bronchiectasis, liver disease, or panniculitis

C-ANCA: cytoplasmic antineutrophil cytoplasmic antibody; and AATD: alpha-1 antitrypsin deficiency. *AATD should be diagnosed by genotyping rather than by determination of the serum concentration of AAT.

Given that AATD is inherited in an autosomal codominant manner, testing should be offered to first-degree relatives of index cases, even if those relatives are asymptomatic, because of the risk that they could have some variant allele or even AATD.^11^ Family members who carry mutations benefit from genetic counseling and preventive measures, the most important of which are avoiding smoking and exposure to environmental or occupational pollutants.^76^ For example, the risk of developing COPD has been shown to be 5-10 times greater for smokers who are heterozygous for the Z allele and another normal allele (i.e., with the Pi*MZ genotype) than for smokers with the Pi*MM genotype.^31^ However, among nonsmokers, the risk of developing COPD is similar between those two genotypes.^79^ When evaluating family members, the determination of serum AAT concentrations is not recommended as the initial test, because it does not detect the genetics of the alleles; a patient with normal or near-normal serum concentrations of AAT could mistakenly be categorized as genetically normal. Therefore, family members should always be initially evaluated by genotyping.^2^ For genetic counseling purposes, the partner of an individual with the Pi*ZZ genotype or with some rare or heterozygous association should also be tested. Because the Pi*MZ phenotype is not uncommon and marriages occur by affinity, it is advisable to test the children of a couple who are both carriers of the Pi*MZ genotype, in order to identify those with a severe homozygous genotype.^9^ The current guidelines do not recommend testing the general population, adolescents, or newborns.^78^ ^,^ ^81^

### Diagnostic methods

#### Quantitative measurement of AAT

The measurement of serum AAT concentration is preferably performed by immunonephelometry, because of the high sensitivity of that method. The serum concentration can be expressed in milligrams per deciliter (mg/dL) or millimoles per liter (µmol/L or µM), with normal values of > 113 mg/dL and 20-39 µM, respectively. Values below 110 mg/dL indicate the possibility of a mutant allele (S, Z, or a rare one) and serve as a guide to continue investigating the genotype. Values ≥ 11 µmol/L are considered protective. In patients with lung or liver disease, below-normal serum AAT values should represent a warning sign for further investigation. In general, approximately 80% of serum AAT reaches the interstitial fluid and 10% reaches the epithelial lining fluid.^82^ The total volume of serum AAT is second only to that of plasma albumin.

Because AAT is an acute phase protein and its concentration can be increased by 75-100% in patients with infection or inflammatory disease,^83^ serum AAT concentrations should be measured when the patient is in a stable phase. That change is more evident at intermediate concentrations of AAT. It is recommended that the level of C-reactive protein be measured because a high level indicates the possibility of an ongoing infection, in which case the AAT test result should not be taken into account and the test should be repeated.^12^ The concentration of AAT can also be abnormally high in the third trimester of pregnancy,^84^ in very elderly individuals,^85^ and in women who routinely use oral contraceptives.^86^ Conversely, low AAT concentrations can be associated with hypoproteinemia and liver failure. It has been observed that the median AAT concentration decreases in the first six months of life and increases to adult concentrations by the end of the first year.^87^ Data related to serum AAT levels in blood samples are available for most of the states in Brazil.

Serum AAT concentrations can also be measured by DBS sampling, which consists in collecting a drop of blood by pricking the distal region of one of the fingers with a lancet or by performing venipuncture. The blood should be distributed evenly across the five circles of a filter paper (Whatman 903; Sigma-Aldrich, Burlington, MA, USA), ensuring that the blood soaks through to the back of the card. If the blood is collected by venipuncture, 50 µL of blood can be distributed evenly across the circles with a pipette. Thereafter, the filter paper should be dried in room air for 12 h and stored in an envelope, away from humidity and light.

### Qualitative measurements of AAT

#### AAT phenotyping

In the diagnosis of AATD, phenotyping is performed by electrophoresis with isoelectric focusing, observing the migration of the alpha-1 protein at pH 4.0-5.0. Although this differentiation method is not difficult to perform, it is quite time-consuming and requires great experience on the part of the technician because the different mutations migrate at very similar speeds due to small changes in the electrolytic dissociation constant.^88^ Null (Q0) alleles do not produce alpha 1 and are not recognized on electrophoresis.

#### Genotyping

Genotyping identifies allele variants that occur due to mutations in the *SERPINA1* gene locus. Analysis of the DNA region using polymerase chain reaction amplification allows the identification of variants. Genetic analysis can be performed in tissue cells, in white blood cells, or in blood samples collected on filter paper (by DBS sampling).

A new AAT genotyping method in which cells are collected from the oral mucosa (by swab), developed by Progenika Biopharma (Derio, Spain) and widely used in Brazil since 2018, allows the simultaneous identification of the 14 most common variants.^89^ The collection of cells from the cheek mucosa by swab is minimally invasive, is rapid, and does not require drying time; the specimen remains stable for two months in room air and can be transported by regular mail.^90^ The nondetection of any of the 14 mutations on the test is reported as “variant not detected”, which translates to a 99% chance that the subject has the MM genotype.^91^

#### Genetic sequencing

If no mutant allele is detected and the blood concentration is below 50 mg/dL or if there is discordance between the serum AAT concentration and the genotype, genetic sequencing is necessary.^2^ ^,^ ^9^

### Algorithm for diagnosing AATD

**Figure 1:**
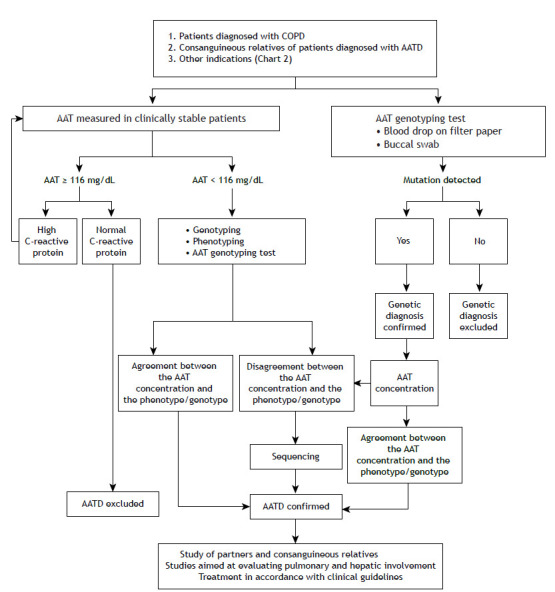
Diagnostic algorithm for alpha-1 antitrypsin deficiency (AATD). In cases of patients diagnosed with AATD, investigate partners to assess the risk of the disease in offspring. Measurement of AAT in serum should be performed by nephelometry. For other techniques, apply a conversion factor. If there is high clinical suspicion of AATD, measure the AAT concentration when the patient is in a stable clinical condition.

There are two approaches to the diagnosis of AATD (Figure 1): one begins with the serum concentration of AAT (the conventional approach); and the other begins with phenotyping or genotyping (the alternative approach). In Brazil, measurement in blood samples in more widely available and it is therefore more common to begin with the assessment of serum AAT concentrations. If the serum AAT concentration assessed by nephelometry is below 113 mg/dL, there is a possibility of AATD and genotyping should be requested. If the decision is made to start with genotyping, one alternative is the Progenika Biopharma test with buccal swab collection, which allows the simultaneously identification of the 14 most common variants (Chart 3). In this approach, if any variant allele is present, it is necessary to determine the serum AAT concentration. In either approach, gene sequencing (the most sensitive confirmatory test) may be necessary if the results of the serum screening and genotyping/phenotyping are discordant.

**Chart 3:** Allelic variants of the serine protease inhibitor gene, detected through genotyping.

Variant	Associated allele(s)	Predicted AAT activity
c.187C>T	Pi*I	Reduced (mild)
c.194T>C	Pi*M_Procida_	Reduced (severe)
c.226_228delTTC	Pi*M_Malton_, Pi*M_Palermo_, Pi*M_Nichinan_	Reduced (severe)
c.230C>T	PPS_Iiyama_	Reduced (severe)
c.552delC	Pi*Q0_Granite Falls_	None (absent)
c.646+1G>T	Pi*Q0_West_	None (absent)
c.721A>T	Pi*Q0_Bellingham_	None (absent)
c.739C>T	PI*F	Reduced (mild)
c.839A>T	Pi*P_Lowell_, Pi*P_Duarte_, Pi*Q0_Cardiff_, Pi*Y_Barcelona_	Reduced (mild)
c.863A>T	Pi*S	Reduced (mild)
c.1096G>A	Pi*Z	Reduced (severe)
c.1130dupT	Pi*Q0_Mattawa_, Pi*Q0_Ourem_	None (absent)
c.1158dupC	Pi*Q0_Clayton_, Pi*Q0_Saarbruecken_	None (absent)
c.1178C>T	Pi*M_Heerlen_	Reduced (severe)

AAT: alpha-1 antitrypsin.

Filter paper collection (DBS sampling) has the advantage of being a simple technique, as well as allowing easy storage and transport of the samples to a central laboratory for the measurement of serum concentration and genotyping/phenotyping. However, the disadvantage is that there are very few laboratories in Brazil that handle the analysis of DBS samples.^12^

## MONITORING OF ASYMPTOMATIC PATIENTS

It is not uncommon for low serum concentrations of AAT not to result in functional or anatomical changes that manifest as respiratory symptoms. This poses a clinical challenge: how should we manage asymptomatic cases of AATD? Although the proportion of individuals with AATD who develop lung disease is still unknown, studies indicate that up to 50% of nonsmokers with AATD maintain normal lung function throughout life.^9^

The first step is an adequate assessment of the symptoms. In patients with progressive lung disease and a gradual loss of function, the symptoms may be underestimated. To avoid discomfort, the patient might reduce their activities of daily living, attributing their symptoms to aging or a sedentary lifestyle. Objective questionnaires such as the Medical Research Council dyspnea scale, COPD Assessment Test or London Chest Activity of Daily Living scale can help identify limitations in patients who do not objectively report symptoms during the history taking.^92^

Patients should be advised to avoid exposure to factors that can increase the risk of emphysema. All smokers should receive smoking cessation counseling and treatment. In addition, it is important to assess and provide guidance on environmental and occupational exposure to smoke and toxic fumes, because eliminating such exposure could slow the progression of lung disease in patients with AATD.^93^

It is essential to monitor patients with AATD for the appearance of pulmonary emphysema and liver disease. All patients with severe AATD should undergo pulmonary function tests, unenhanced chest CT, and liver evaluation. Evaluation of liver involvement is especially important in patients with the ZZ, SZ, or rare genotypes that can be risk factors, such as M_Malton_ and S_Iiyama_.^2^ Such patients should undergo periodic evaluations (depending on the clinical presentation), with a physical examination focused on signs of liver disease, liver ultrasound or elastography, and laboratory tests to determine the levels of glutamic oxaloacetic transaminase, glutamic pyruvic transaminase, alkaline phosphatase, gamma-glutamyl transferase, albumin, and bilirubin, as well as coagulation tests.^13^ In patients with AATD, liver fibrosis can be silent and hepatocellular carcinoma occurs in 3%,^94^ the risk factors being male sex, age over 50 years, lifestyle, and persistently elevated liver function values.^12^

The best frequency of monitoring to detect the onset or progression of lung disease in patients with AATD has yet to be established and depends on serum AAT concentration and pathogenicity of the mutation presented. Generally, in patients without respiratory symptoms and with normal baseline spirometry findings (i.e., FEV_1_ ≥ 80% of predicted), spirometry is repeated every 12 months or every 6-12 months if the symptoms change. If possible, it is important to measure volumes, flows, and DL_CO_ at the beginning of follow-up. In comparison with the decline in FEV_1_, the change in DL_CO_ occurs earlier and is more intense. It is essential that an asymptomatic patient with a mutation be advised to seek medical assistance if they notice dyspnea, especially on exertion, or any other significant lung impairment, such as pneumonia. An unexplained reduction in post-bronchodilator FEV_1_ to below the lower limit of normal is a sign to consider starting specific treatment.^2^

Monitoring of asymptomatic patients with AATD should be personalized. The pathogenicity of the specific mutation and serum concentrations are factors to be considered when establishing the frequency of monitoring. Genetic counseling and lifestyle guidelines to avoid potentially harmful exposures are the most important measures in this context. It is essential to consider a multidisciplinary approach, involving pulmonologists, hepatologists, geneticists, and other specialists, to ensure the best quality of life and prognosis for these patients.

## GENERAL OR NONSPECIFIC TREATMENT

Treatment for AATD includes behavioral and pharmacological therapy for smoking cessation, together with guidance on the importance of avoiding exposure to irritants (tobacco smoke, dust, smoke, pollutants or aerosolized chemicals). Other therapies are similar to those indicated for patients with COPD not caused by AATD: anti-influenza and anti-pneumococcal vaccination; pharmacological treatment, as recommended by the GOLD,^11^ including the use of slow-release bronchodilators (muscarinic antagonists and β_2_ agonists) and inhaled corticosteroids (in special situations); pulmonary rehabilitation; and nutritional support. Exacerbations should be treated, and when they become recurrent, the use of prophylactic antibiotics, inhaled corticosteroids, or both is indicated. Patients who meet the relevant blood gas criteria should be referred for supplemental oxygen therapy.^75^

Surgical treatment is also indicated for some patients with AATD and pulmonary emphysema. The results are limited and not necessarily promising. In very well-selected cases, lung volume reduction surgery has produced positive results (functional improvement and better quality of life).^95^

At major centers worldwide, AATD is one of the main indications for lung transplantation. It has been well documented that lung transplantation results in improved quality of life and survival in patients with AATD.^96^ ^,^ ^97^

## SPECIFIC TREATMENT

Patients who are receiving optimized COPD treatment should also undergo AAT replacement therapy, which should be personalized.^11^ It is recognized that there are patients with severe mutations in whom lung function and clinical status remain stable.

Several studies have shown that health professionals have limited knowledge about AATD and its diagnosis, as well as that there is significant inequality in access to specialized care and treatment.^9^ The Organic Health Laws of the Brazilian Unified Health Care System provide for the right to the promotion, protection. and recovery of health for every citizen, although it is very difficult to provide care for rare diseases and AATD is no exception.

Although there have been recent improvements in awareness of AATD and in the understanding of its treatment, there are still challenges related to improving diagnosis, providing optimal general treatment, and improving access to the specific treatment, intravenous AAT therapy, which is the only available pharmacological intervention that can slow the progression of the disease.^98^ Interpreting genetic variants and their importance, understanding the role of patient and family screening, and the management of the disease all require expertise and seem to be best developed at reference centers that have the capacity to provide appropriate care and treatment, including intravenous AAT replacement therapy.^1^ ^,^ ^9^

Intravenous replacement therapy with purified AAT is the mainstay of treatment for AATD, although lung volume reduction surgery and lung transplantation are of real value in selected cases. To avoid or reduce the destruction of lung parenchyma by AATD is the primary goal of AAT replacement therapy,^99^ given that intravenous augmentation by infusion of combined human alpha-1 trypsin inhibitor is the most direct, efficient, and unique means of increasing AAT concentrations in the blood and lung interstitium and of preventing the progression of emphysema to a more severe form.^100^

There are several guidelines and scientific articles with recommendations on the treatment of AATD in adults (Chart 4). In Europe and the United States, various studies have emphasized the need for careful diagnosis and access to intravenous AAT replacement therapy in patients with severe AATD. However, there is still a need to improve the biomarkers of emphysema progression and of the response to replacement therapy. Studies on the rate of decline in lung density indicate that this tool is useful in helping to assess the results of replacement therapy. With regard to specific therapy, research on personalized replacement, with individualized selection of the therapeutic regimen, is essential.^9^

**Chart 4:** Criteria for specific treatment of alpha-1 antitrypsin deficiency.

Guideline or summary of product characteristics (country/institution of origin)	Criteria and recommendations
Argentina	Age > 18 years, nonsmoking patients with an AAT < 50 mg • dL^−1^, emphysema on CT, or pulmonary function with an FEV_1_ < 80% of predicted
	No discontinuation of AAT replacement even if FEV_1_ falls below 25% of predicted
	Not recommended in patients with the Pi*MZ genotype or in most patients with the Pi*SZ genotype unless they have an AAT < 50 mg • dL^−1^ and meet the other criteria
Belgium	Nonsmoking patients with an AAT < 50 mg • dL^−1^ and FEV_1_ 30-60% of predicted and a decline in FEV_1_ (% of predicted) > 0.5% per year
	Patients with an FEV_1_ 60-80% of predicted if the decline is > 1% per year
Canada	Nonsmoking patients with COPD (FEV_1_ 25-80% of predicted) attributable to emphysema and serum AAT < 11 µM
United States	Severe AATD in individuals with an FEV_1_ < 65% of predicted
	In patients with a FEV_1_ > 65% of predicted, discussion of benefits and costs
Spain	Age > 18 years, nonsmoking patients with an AAT < 50 mg • dL^−1^, emphysema on CT or pulmonary function with an FEV_1_ < 80% of predicted
	No discontinuation of AAT replacement even if FEV_1_ falls below 25% of predicted
Portugal	Age > 18 years, COPD attributed to emphysema caused by AATD, serum AAT < 57 mg • dL^−1^, FEV_1_ 30-70% of predicted, or if the FEV_1_ is > 70% of predicted, a decline in FEV_1_ > 120 mL • year^−1^
	Case-by-case decision in other cases and no discontinuation of AAT replacement in case of deterioration of lung function
Poland	Severe AATD, emphysema, nonsmoking patients with an AAT < 11 µM, FEV_1_ 30-65% of predicted or a decline in FEV_1_ > 50 mL • year^−1^
BRAZIL/BTA	Age > 18 years, nonsmoking patients, preferably treated at reference centers, with severe deficiency (serum AAT < 57 mg • dL^−1^ or < 11 µM), with proven functional loss above the physiological level (even in patients with normal lung function or with mild, moderate, or severe airflow obstruction)
	For patients with an FEV_1_ > 80% of predicted, excessive functional loss defined as > 100 mL/year over a three-year period
	No discontinuation of the specific treatment even if FEV1 falls below 25% of predicted

AAT: alpha-1 antitrypsin; AATD: alpha-1 antitrypsin deficiency; BTA: Brazilian Thoracic Association.

Adapted from Miravitlles et al.^33^

Some guidelines, such as those developed in Belgium,^101^ Portugal,^13^ Poland,^102^ and Canada,^103^ have introduced the drop in FEV_1_ (% of predicted) after bronchodilator use as a criterion for initiating treatment in patients with AATD, although the drop in FEV_1_ currently has less sensitivity than does the decrease in lung density. There seems to be a consensus that intravenous AAT replacement therapy is indicated in nonsmokers or former smokers over 18 years of age with a genetic variant consistent with severe AATD, a low serum AAT concentration (< 11 µmol/L or < 57 mg/dL), and evidence of airflow limitation on pulmonary function testing.^5^ ^,^ ^11^ ^,^ ^13^ ^,^ ^101^ ^-^ ^103^ Identifying an accelerated decline in lung function on the basis of the drop in FEV_1_ in serial functional assessments is a more sensitive method than is the identification of isolated airflow obstruction, because the former can indirectly indicate lung tissue destruction due to enzymatic imbalance. Serum AAT concentrations above 11 µmol/L or 57 mg/dL are considered protective and should be used as a parameter to determine the effectiveness of replacement therapy.^104^

Some patients present emphysema on chest CT without airflow limitation on spirometry, demonstrating that chest CT is more sensitive, at least initially, than is a functional assessment. In patients suspected of having developed emphysema, annual spirometry should be used in order to monitor lung function. A real loss of lung function is defined on the basis of the change over a three-year period, a decline in FEV_1_ ≥ 100 mL/year being considered excessive and an indication for the initiation of specific therapy.^5^ ^,^ ^11^ ^,^ ^13^ ^,^ ^101^

The “Intravenous augmentation treatment and lung density in severe a1 antitrypsin deficiency” study and its open-label extension, designated the RAPID and RAPID-OLE studies, respectively,^105^ ^,^ ^106^ were the largest clinical trials of intravenous AAT replacement therapy ever conducted. The RAPID study was a placebo-controlled trial in which patients were randomly assigned to receive, on a weekly basis, either intravenous AAT (60 mg • kg^−1^) or placebo, for 24 months.^105^ The RAPID-OLE study began at that point: patients who had been receiving the placebo began to receive the AAT replacement, while those who were already receiving AAT replacement continued to receive it.^106^ In the RAPID study, at the end of the 24-month period, there was a reduction in grams of lung tissue per liter of lung volume (g/L) of 2.90 g/L in the group receiving AAT replacement, compared with 4.38 g/L in the placebo group, a statistically significant difference. In the RAPID-OLE study, at the end of a second 24-month period (i.e., at 48 months after the start of the trial), those who continued to receive AAT replacement had lost 5.03 g/L, whereas those who had received placebo for 24 months and the received AAT replacement for 24 months had lost 6.32 g/L. During the RAPID study (between day 1 and month 24), the annual loss of lung tissue, measured in TLC, was 33% greater in patients who were receiving placebo. During the RAPID-OLE study (between month 24 and month 48), the rate of loss in those who started receiving AAT replacement after month 24 decreased and, by month 48, was equal to that of those who received AAT replacement from the beginning, although their lung density was still lower than was that of those who had received AAT replacement from the beginning of the trial. Therefore, the efficacy of AAT treatment was maintained throughout the 48-month trial period in the group receiving AAT replacement. The two studies clearly showed two important points^105^ ^,^ ^106^: during the first 24 months, AAT replacement protected the lungs from excessive loss of lung tissue; and, by the end of month 48, the group that had previously received placebo and started receiving AAT replacement had a loss rate equal to that of the group that had received AAT replacement from the beginning of the study, but it did not return to the previous value, indicating that the loss was permanent. These two studies leave no doubt about the effectiveness of specific treatment with intravenous AAT replacement and increase the certainty that the therapy truly slows the progression of lung destruction.

For more than 30 years, there has been evidence of the biochemical efficacy of intravenous AAT replacement therapy, with increased concentrations in serum and in the lungs, including the epithelial lining fluid. In 1989, US Food and Drug Administration approved AAT purified from human plasma for intravenous administration, at a dose of 60 mg/kg, administered weekly.^107^ Its administration is contraindicated in patients with selective IgA deficiencies, because of the possibility of severe anaphylaxis. In the 13 years that intravenous AAT replacement therapy has been employed at the Hospital Regional da Asa Norte in Brasília, in 8-12 patients per week, there has never been an anaphylactic reaction. Although the incidence of severe IgA deficiency (< 7 mg/dL) is very low (< 1%) in the general population, we recommend measuring the total IgA concentration before starting replacement therapy, given that all commercial preparations for AAT replacement therapy contain some IgA and patients with severe deficiency are at risk of anaphylaxis after infusion of pooled human AAT. The AAT should preferably be administered at reference centers and by trained staff.^1^ ^,^ ^9^ ^,^ ^108^ There is insufficient evidence to recommend the use of biweekly or monthly applications, although that is left to the discretion of the reference centers, according to the condition of the patient. The ongoing ProlAstin-c Randomized Therapy with Alpha-1 augmentation trial is evaluating weekly AAT replacement in three arms-60 mg/kg, 120 mg/kg, and placebo-with the hypothesis that the 120 mg/kg dose would provide a higher level of protection (> 20 µmol) and less lung tissue loss over three years.^109^

It is known that AAT can exert effects beyond protease inhibition, having antibacterial effects by inhibiting pro-inflammatory responses induced by bacterial endotoxins *in vitro* and *in vivo*.^110^ Treatment with AAT replacement is associated with a reduction in the concentration of leukotriene B4, one of the most important mediators of neutrophil recruitment and activation, with a central role in airway inflammation.^111^

Treatment with AAT replacement has an indefinite duration, is costly, and requires careful consideration. The data supporting the clinical efficacy of intravenous AAT replacement are robust and include randomized trials that evaluated outcomes such as serum AAT concentrations, lung function, and lung density on chest CT.^3^ ^,^ ^9^ ^,^ ^105^ ^,^ ^106^ ^,^ ^111^ ^-^ ^113^

Supported by robust data in the literature, we recommend AAT replacement for any patient over 18 years of age with severe AATD; that is, serum concentrations below the protective level (< 57 mg • dL^−1^ or serum AAT < 11 µM), with proven functional loss above the physiological level (even if lung function is still normal or if there is mild, moderate or severe airflow obstruction). For patients with an FEV_1_ > 80% of predicted, functional loss is considered excessive if it is > 100 mL/year in annual assessments over a three-year period. Short-term variations may occur due to test-retest variability. The specific treatment should not be discontinued even if the FEV_1_ falls below 25% of the predicted value. There is no consensus on AAT replacement in patients with the Pi*SZ genotype, even in those with serum AAT concentrations < 11 µM or < 57 mg • dL^−1^. It is possible that the EARCO data will answer this question.

During the period of intravenous AAT replacement, routine monitoring of serum AAT concentrations is not recommended.^9^ Lung function should be monitored annually, except in patients with a suspected decline above the physiological level, in whom functional assessment should be performed as needed. The objective of monitoring lung function is to assess its decline and estimate the prognosis. It is interesting to use questionnaires to assess quality of life, assess COPD, and monitor exacerbations.^9^ ^,^ ^105^ Despite the cost, AAT replacement is a specific treatment that will slow the destruction of the lung parenchyma, consequently increasing survival,^108^ and should therefore be offered to all those who need it.

In addition to the Pi*ZZ, Pi*Q0, and Pi*SZ alleles (when accompanied by serum concentrations below the protective level), other genetic variants can lead to low AAT concentrations and the development of emphysema requiring AAT replacement: Pi*M_Procida_, Pi*M_Malton_, Pi*M_Palermo_, Pi*M_Nichinan_, Pi*S_Iiyama_, Pi*Z, and Pi*M_Heerlen_. Several variants can also cause severe liver disease^12^: Pi*M_Malton_, Pi*M_Palermo_, Pi*M_Nichinan_, Pi*S_Iiyama_, Pi*P_Lowell_, Pi*P_Duarte_, Pi*Q0_Cardiff_, Pi*Y_Barcelona_, Pi*Z, Pi*Z_Augsburg_. All patients with genetic variants that can cause severe liver disease should also receive specialized hepatology care.

## RECOMMENDATIONS

All adults with persistent post-bronchodilator airflow obstruction on spirometry should be tested for AATD. In addition, testing outcome measures include emphysema in individuals under 45 years of age, emphysema in a nonsmoker or smoker with a minimal smoking history, emphysema that is predominantly in the basal regions on chest CT, a family history of emphysema or liver disease, current or previous panniculitis, and current or previous unexplained chronic liver disease.^3^ ^,^ ^9^ ^,^ ^11^

Given that AATD is inherited in an autosomal codominant manner, first-degree relatives (parents, siblings, children, and partners) of the index case should be tested, even if they are asymptomatic, because there is a risk that they will present some variant of AAT.^11^ If the variant identified in a relative is not severe, it is not necessary to measure the serum concentration of AAT, although it should always be measured in relatives carrying severe mutations.^76^ The risk of developing COPD is 5-10 times greater in smokers who are Pi*MZ heterozygotes than in smokers with the Pi*MM genotype.^31^ However, among nonsmokers, the risk of developing COPD is comparable between those two genotypes. The same applies to heterozygous carriers of any rare severe variant.^79^

Individuals who are heterozygous for Pi*SZ, for two very rare variants, or for Pi*Z and another rare variant should be monitored and advised to avoid risk factors for COPD. In symptomatic individuals with an AAT test showing no variants, genetic sequencing should be requested, because they might be carriers of a null variant, in which case, serum AAT will be undetectable.^2^

An individual with the Pi*MZ genotype or with the Pi*M genotype in combination with a rare allele requires special attention, because if their partner also has the Pi*MZ genotype, the couple could produce a child with the Pi*ZZ genotype.^9^ In such cases, the person with the Pi*MZ or with the Pi*M genotype and a rare allele should be advised that if they have a child, the genotype of that child should be assessed and if homozygous for severe deficiency, the child should be advised not to smoke and to avoid other risk factors.

After genotyping and serum measurements have been performed on the family members of the index case, a meeting should be held with the entire family to explain what AAT is, where it is produced, its action, what normal and variant alleles are, and the possible consequences for individuals with AATD who are carrying variants, as well as the risk factors for lung lesions. These meetings can be held virtually; the purpose is to clarify, guide, and, above all, reassure the family.

To avoid stigmatization of AATD carriers, it is necessary to guide the individual to lead a healthy life, to not feel limited, and to not fear the future. In relation to the discovery of children with variants that may predispose to lung lesions, parents should be advised not to smoke, to educate children not to smoke or expose themselves to risk factors, and to explain the genetics of AATD only after they are old enough to understand the facts. Parents should guide children to have as normal a life as possible so that they do not feel limited.

Current guidelines do not recommend testing the general population, adolescents, or newborns.^78^ ^,^ ^81^ Genotyping adolescent and young smokers and advising them to quit smoking could be an important preventive measure. Although that measure has never been implemented in any country, it could be cost-effective.

Finally, it is possible to group individuals with AATD into a database and monitor them, which allows us to understand the natural history of the disorder, provide earlier interventions, personalize treatments, and provide family genetic counseling. The international EARCO database currently includes anonymized data provided by researchers in 24 European countries and the Americas (Argentina, Colombia, Costa Rica, and Canada). Data storage partnerships with the EARCO can be established only by individual hospitals and not by a group of centers representing a country or a medical society. Although patient data are considered the property of the hospital and the principal investigator, they can be shared if authorization is obtained from the hospital coordinator of the research group.

When AAT replacement is indicated, it should follow the criteria described in this document.^1^ ^,^ ^9^ ^,^ ^108^ ^,^ ^114^ Experience with lung volume reduction surgery in patients with AATD is limited. Lung and liver transplantation are reserved for severe and terminal cases of AATD.^96^ After liver transplantation, AATD is corrected because the phenotype-normal donor liver produces and secretes AAT.
